# "The Hospital" Nursing Mirror

**Published:** 1900-03-10

**Authors:** 


"The Hospital" Nursing Mirror.

Being the Nursing Section of "The Hospital."

[Contributions for this Section of "The Hospital" should be addressed to the Editor, The Hospital, 28 & 29, Southampton Street, Strand, London, W.C., and should have the word "Nursing" plainly written in left-hand top corner of the envelope.]

### Notes on News from the Nursing World.

### THE PRINCESS OF WALES'S WAR FUND.—LETTER FROM HER ROYAL HIGHNESS.

A cheque for £231, the amount contributed by nearly 1,800 nurses through The Hospital newspaper to the Princess of Wales's War Fund, has been sent to Her Royal Highness, and the following letter in acknowledgment has been received by Sir Henry Burdett:—

"Marlborough House, Pall Mall, S.W.,

"March 4th, 1900.

"Dear Sir Henry,—I am desired by the Princess of Wales to request you to convey the expression of Her Royal Highness's warmest and most sincere thanks to the 1,779 nurses from all parts of the Empire for the sum of £231 which they have so kindly and generously subscribed to the Princess's War Fund. They may rest assured that H.R.H. will spare no pains to lay out the money in the way best calculated to alleviate the sufferings and add to the comforts of the gallant troops who are fighting so bravely for their Queen and country in South Africa.—Believe me, yours sincerely, "Charlotte Knollys."

The gracious and appreciative language used by the Princess will, we are sure, afford great pleasure to every contributor to the Fund, and each nurse will regard the letter in the light of a personal acknowledgment from Her Royal Highness that she has done something "to alleviate the sufferings and add to the comforts" of the soldiers, for whom the Princess herself has shown such practical and continuous sympathy.

### NURSES AND ORDERLIES AT THE BASE HOSPITALS.

Mr. Frederick Treves, in one of his interesting letters, mentions that Miss McCaul and two Netley sisters have rendered invaluable service in nursing the wounded after the Battle of Spion Kop. He says that they worked day and night, and "it is hard to imagine what we should have done without them." Mr. Treves adds, "three nursing sisters can hardly cope with a hospital containing over 700 wounded men, and the great mass of the work falls upon the orderlies, who are a singularly efficient, obliging, hard-working set of men." This statement is particularly interesting when read in the light of an interview reported in another column with Miss Norman, the Lady Superintendent of Netley Hospital. Miss Norman shares the good opinion of Mr. Treves in reference to the orderlies, and states that 10 or 11 sisters are sufficient for nursing purposes at Netley. But, as she points out, patients at Netley largely consist of practical convalescents; whereas the patients in the base hospitals must for the most part be more or less serious cases. The whole tenor of the account of nursing at Netley goes, in fact, to prove that the idea of three sisters in a hospital attending on 700 wounded soldiers is perfectly absurd. This is a question that imperatively needs the attention of the War Office authorities. Whatever may be the cogency of the arguments in favour of the relatively small supply of sisters in the military hospitals at home, there cannot be any doubt whatever that a much larger proportion is urgently necessary to nurse acute cases in South Africa. As a concrete example, take the 800 cases of typhoid fever which are stated to be lying ill at the present time in Ladysmith. How many nurses would be employed in England to nurse this number of cases? The average number of patients, whether at Charing Cross, the Royal Free, St. George's, St. Thomas's, the North-West London, King's, the London, or Guy's Hospitals run from 2·3 to 2·6 per nurse, and if the typhoid patients at Ladysmith were to be nursed according to the London standard 320 nurses would be at once required there alone. It is then, in our opinion, childish to pretend that no more nurses are required at the seat of war.

### LADY O'HAGAN AT THE FRONT.

Amongst those who are utilising our special gifts and opportunities for the amelioration of the wounded soldiers in South Africa is Lady O'Hagan. She accompanied her son, an officer in the Grenadier Guards, to Cape Town, and, when he joined Lord Methuen at Modder River, she obtained the sanction of Lord Roberts to proceed to Naauwpoort with a small party of nurses to render such services as may be required. Lady O'Hagan secured her staff from the Queen's nurses at Cape Town, and has taken with her all supplies, including hospital tents. She has long been specially interested in nursing work at home, and is President of the Workhouse Infirmary Committee of Burnley, and also a member of the Board of Management of the Burnley Victoria Hospital.

### THE MATRON OF THE SCOTTISH SOUTH AFRICAN HOSPITAL.

A correspondent states that the matron of the hospital which is to be equipped at the expense of the Scottish people, is "to be a lady who, though much respected on personal grounds, does not hold a three years' certificate of any recognised training school." She inquires why the nursing department of the hospital is not to be officered by the best of Edinburgh nurses, and says "it will be difficult to get fully certificated surgical nurses to work under a matron not properly qualified." If an appointment of the character indicated by our correspondent is in contemplation, it would, we think, be a mistake; but it will probably be found that a choice which commands the confidence of trained nurses will be made.

### TO "NIGHTINGALE" NURSES.

Many of the Nightingale nurses have expressed a desire to join in sending an address of congratulation to Miss Nightingale on the occasion of her approaching birthday, May 12th, 1900, when she will be 80 years of age. Any Nightingale nurse who wishes to have her name attached to the address should write to Miss Gordon so as to be received not later than April 30th. Address The Matron, St. Thomas's Hospital, Palace Road, London, S.E.

### CHANGES IN THE INDIAN ARMY NURSING SERVICE.

An exchange of stations has been sanctioned between Senior Nursing Sisters Harris and Hislop, the former proceeding to Quetta. The principal medical officer of her Majesty's Forces in India has directed Lady Superintendent Moore, Indian Army Nursing Service, to proceed to Poona at once and assume the duties of Superintendent I.A.N.S., Bombay Command. Miss Moore will be relieved of her duties at Rawal Pindi by Sister Loch. Miss S. H. B. Leslie has been admitted to the rank of nursing sister in the Indian Army Nursing Service. Miss Dora Angelo has been appointed Staff Nurse, Ripon Hospital, Simla, in succession to Miss Penny, who has retired. It is proposed to establish an association of male nurses in Calcutta and Simla, and to recruit the ranks of the association chiefly from reserve men of the Royal Army Medical Corps, who have had experience of tropical nursing in the colonies.

### ST. GEORGE'S INFIRMARY.—A NEW DEPARTURE WITH A VENGEANCE.

It will be seen from a letter (printed on page 305) by a nurse at St. George's Infirmary, Fulham Road, that the Guardians of St. George's, Hanover Square, have made a new departure. As our readers are aware, it is no easy matter to induce these gentlemen to institute changes, and though to some extent, thanks to our correspondent, who first drew attention to the need for reform at this infirmary, we have succeeded in effecting some little improvement, the intended erection of a nurses' home is pleaded as a sufficient reason for delaying further alterations. Meanwhile, for some cause which is not explained, every member of the staff at St. George's Union has been called upon to sign the document mentioned by one of their number; of course, the Guardians are acting within their legal rights, but the course they are pursuing is not customary, and is distinctly calculated to increase the difficulties experienced in obtaining good nurses at workhouse infirmaries. It is not fair that a nurse contracting an infectious disease whilst in the discharge of her duty to the Guardians should have to appeal for refuge to her friends; and the idea that she should be compelled to submit herself to a medical examination to "any doctor whom the Guardians may appoint for that purpose at any time" is abominable, especially as the doctor is to report the result of his investigations to the Guardians, but we are quite certain of this, that no course could have been adopted more calculated to deprive the Guardians of the services of self-respecting women of every class. A medical examination is a term without limitation, and such a consent may be used to enforce on unfortunate nurses such a humiliation as was at the very root of all the protests against the C. D. Acts, and we protest in the strongest manner against placing such a weapon in the hand of any official or board of guardians. As to whether a nurse is justified in refusing to sign such a document, this must depend upon whether she is prepared to take the risk of dismissal. Probably the Guardians will be materially influenced by the manner in which the staff treat the demand.

### ATTACK BY A GUARDIAN OF ST. GEORGE'S UNION ON THE MATRON AND NURSES.

At a meeting of St. George's Board of Guardians last week a recommendation by the Visiting Committee that 10 additional probationers should be appointed was the subject of discussion. According to the *West London Press* of Saturday, Mr. Everitt made some extraordinary remarks:—

"His inquiries led him to believe that the work which the nurses had to do was not too heavy in character, except in chronic cases of illness, when a great deal had to be done. . . . Ever since the matron had been at the infirmary expenses had been going up. He considered her to be the most extravagant woman he had ever met with in his life. Mr. Everitt went on to state that he had seen a nurse nursing a baby. When he came up she said, 'Oh, dear, dear, what am I to do. I have been nursing this baby three hours.' . . . Then, again, he knew of another nurse at the Redcliffe Gardens home who spent a good deal of her time in spreading butter on the bread for the other nurses. . . ."

We are glad to observe that Dr. Myers severely condemned these unseemly personalities, and said "he considered that Mr. Everitt had thrown a slur upon the medical superintendent and the matron, both of which were undeserved:—

"Mr. Everitt had said that the matron was the most extravagant woman in the world that he had ever met with. No doubt Mr. Everitt had had considerable experience with women, but he (Dr. Myers) could not for the life of him understand why he should make such a remark. It was unfair to make such allegations."

Not only unfair, but unbecoming.

### SOME OF THE OLD RULES OF ST. THOMAS'S HOSPITAL.

In the current number of *St. Thomas's Hospital Gazette*, "the old rules of the hospital" are published. Some of them are curious reading in the present day. Thus, under the heading of "The Duty of the Sister," we find Rule II. enacts that they "be careful there be no playing at cards, dice, or any other games in this house, to give notice to the treasurer or steward, if any offend therein." Rule III. "That they wash, or cause to be washed, all weak people's clouts, without taking money or reward for the same. That they give the medicines as directed; the night medicines by eight o'clock in winter, and nine in summer." Rule IV. "That they appoint some sober patient to crave a blessing and return thanks at every meal; to read at the desk on Sunday, and the rules and orders to be observed by the patients to be read aloud in the ward every Friday morning." As to "the duty of the nurse," what do nurses of the present day think of the following rules? II. "She must stupe as often, and in such manner, all such patients as the doctors and surgeons shall direct, and attend the working of all the vomits." V. "She is to make all the beds on one side of the ward, and to scour and make clean the beds and floors of the whole ward, with the tables and forms, the passage and stairs, and garrets; to assist her, she may take such patients as the sisters shall think fit and able to help her." VI. "She must keep clean scoured the cans for beer, the broth pails, pans, platters, and plates, &c., fouled at dinner." VII. "She must attend the butler at the ringing of the beer-bell, and take with her such patients as are able to carry the beer in safety to the ward, and not suffer such patients to waste or embezzle it by the way, but see that the cans be carried full into the ward; and in like manner at his ringing the bread-bell, she must attend and take the just number of loaves for the patients, who are intitled to it; and also at the ringing of the cook's-bell, she must attend her, and receive from her the exact quantity of provisions that are appointed for each patient."

### CURIOUS INCIDENT IN AUSTRALIA.

The authorities of the Adelaide Hospital seem, according to the *Melbourne Age*, to have a curious idea of a convalescent retreat. It appears that two of the nurses who had been indisposed were sent to "recruit their strength" to the quarantine station at Torrens Island. Seeing that at Torrens Island a patient suffering from plague was being nursed, and the two ailing nurses were expected to render assistance if required, we should not have been surprised if they had preferred to remain at Adelaide. Nor is it astonishing that in the circumstances the report was current that the nurses themselves were suffering from plague. The secretary of the Central Board of Health states, however, that they "have not been quarantined as infected nurses in any sense."

### SCARBOROUGH GUARDIANS AND THEIR NURSES.

The Scarborough Guardians have not yet been able to secure the nurses they require. Having selected two out of 14 applications for two vacancies they supposed they had come to the end of their difficulty, but, when the two nurses had gone over the infirmary and ascertained the nature of the duties they would have to perform, they declined to take up their appointments. The remaining applications have since been considered, and other applicants have been communicated with. But it is probable that until the conditions of service are altered, the Scarborough Guardians will continue to find it no easy matter to obtain nurses whose experience is of the kind they desire.

### RESIGNATION OF NURSES AT A WORKHOUSE INFIRMARY.

We understand that at the Belper Union Workhouse five nurses out of a staff of seven have resigned their positions in the workhouse hospital, giving as their reason for so doing the attitude of the workhouse master towards them. One of the nurses is the superintendent, and the other four are probationers. Three of them were asked to suspend their notices taking effect until there had been inquiry into the cause of their resignation, but they declined to reconsider the position. One of the matters in dispute seems to be nothing more serious than half an ounce of butter or two or three ounces of bread, which the nurses found to be missing in their patients' allowance, and for which they made request. The Guardians instituted an inquiry into the cause of the nurses' resignations, at which the Government inspector was present, but as the inquiry was conducted with closed doors the ratepayers are in ignorance of the result of the inquiry. It is stated that friction of this kind between workhouse master and hospital staff has gone on for 15 years.

### MIDDLESBROUGH NURSES' HOME.

Although the tenth annual report of the Middlesbrough Nurses' Association is in the main of a satisfactory character, we share the regret of the Mayor that the people of the town themselves do not show their appreciation of the work in the manner that might be expected, considering the benefits gained. The Mayor himself, speaking as a poor-law guardian, says that if it were not for the labours of the association there would be an unwelcome addition to the poor-rate —a very practical reason why the support given to the association should be materially increased.

### ST. LUKE'S HOME, VANCOUVER.

In the report of St. Luke's Home, Vancouver, for last year the pleasing announcement is made that the Sons of England Ward, known as the Jubilee Ward, has proved a great boon to members of the order who in their sickness have made use of it. Towards the end of the year it was decided to increase the accommodation for the staff of nurses, and the house next door was taken. There are now, as well as a sister in charge, six nurses and a probationer, and all were kept very busy either at the home or nursing out during the twelve months. Those of our readers who are interested in British Columbia will like to know that not only did the people of that colony send a substantial sum to the Patriotic Fund, but also a large consignment of useful articles, including 3,000 pairs of hand-knit socks, each sock being filled with various comforts such as buttons, laces, needles, and thread, and boracic acid in small packets. Every pair of socks had the giver's name printed or worked on, so that if the Canadian troops get them they will know many of the senders. Besides this consignment, 500 dollars are in the bank until the British Columbians are advised from the front what to do with them.

### SHORT ITEMS.

The Queen has presented to the Helena Nursing Home, Reading, a pretty woollen scarf, knitted by herself in token of the interest she takes in that institution.—The Countess of Derby will lay the foundation-stone of a new home for the nurses of the Blackburn District Association on Thursday, April 26th.—On Tuesday the first of a series of free weekly lectures to nurses at the City Orthopædic Hospital was given by Mr. John Poland on "The Human Skeleton." The series will deal with elementary anatomy and physiology, nursing, application of splints and bandages, and antiseptic dressing. The other lecturers are Messrs. Chisholme Williams, Noble Smith, Jackson Clarke, and Henry Davis, and the lectures commence at half-past five.—Miss Florence Baxendale has written, under the title of "The Disenchantment of Nurse Dorothy," a new novel, giving what purports to be a vivid and truthful account of a nurse's life in the hospitals of to-day. It will be published immediately by Messrs. Skeffington.—An entertainment of an excellent character was given at the Cancer Hospital, Fulham Road, to the in-patients of this institution on Thursday evening, entitled "Our Boys," by the Socials Dramatic Company, under the direction of Mr. Reginald H. Lindley.—Miss Mary H. Kingsley, in denying that she is going out in the capacity of nurse to one of the Army hospitals in South Africa, says: "What makes the statement more trying is that I am one of those people keen on seeing nursing raised to the full level of a profession, with all nurses who undertake that work as a profession registered and certificated, and I personally have no certificate; it is not my profession; I only do it when the 'regulars' are not available."—Miss Edith Rhodes, sister of Mr. Cecil Rhodes, sails on Saturday week for South Africa. She intends to visit most of the military hospitals, and will have the opportunity not only of taking with her more good things for the sick and wounded soldiers, but also of seeing how much those already sent have been appreciated.—The hospital ship "Sunrise" fitted out by Mr. Jesse Coope, the well-known brewer, is doing useful work in South Africa as a tender to the "Nubia," and two nurses from the London Hospital are on board.

### Lectures on Medicine to Nurses.

By H. A. LATIMER, M.D. (Dunelm), M.R.C.S. Eng., L.S.A.Lond.; Consulting Surgeon, Swansea Hospital; Past President of the South Wales and Monmouthshire Branch of Brit. Med. Assoc.; Past President of the Swansea Medical Society; Hon. Life Member of the St. John Ambulance Association, &c.

### LECTURE III.

### DISEASE—FUNCTIONAL AND ORGANIC—PAIN.

IN my last lecture I used the term "functional" disease; it now behoves me to speak of disease generally, and to define the meaning of the word. Literally it means want of ease, and may be taken as signifying a state opposed to health. Now it is apparent to everyone that we are liable to suffer from passing ailments as well as to permanent ones. It is true that the passing ailments may be of considerable duration and of much severity, and that the permanent diseases may produce so little suffering that even their existence may be unknown to those who are affected by them. But these last may exist, notwithstanding the absence of symptoms. "Symptoms" are but the cry of suffering organs, and our recognition of them is largely dependent upon their gravity in the first instance, and upon our receptivity in the second. The greatest difference exists between people and between races in this respect. Some folk are so highly sensitive by nature that they feel very acutely disturbances which others are hardly conscious of. In the course of your work you will often be struck with this fact. Ofttimes the difficulty is to find out how much truth there is in the statements made to you by sufferers—whether there is an affectation of distress, or whether the pain or disturbance is real.

Now I do not doubt that, as far as pain is concerned, there are two facts about it—that the intensity with which it is felt varies very much according to the blunted or heightened sensibility of the nervous system of the sufferer, and that it is greatly increased in severity by the amount of attention which is paid to it. Everyone has been conscious of the truth of this last assertion, for there are few of us who have not experienced its verity in our own cases. Excitement and diversion of thought will minimise or delay the sensation until such time as the attention can be directed to the subject. This fact, I believe, is often brought out during the course of a battle, when the wounded combatants are conscious of very little more than the dull sensation of having received a blow when they have had injuries inflicted upon them which, in calmer moments, would there and then have resulted in great pain and shock. Who has not been surprised by the disappearance of a headache or toothache when some pressing matter, involving much attention and thought, has suddenly arisen to divert the attention, such aches returning and re-asserting themselves when the mind has again been able to resume its ordinary quiet state of thought? This fact, indeed, is at the bottom of many of the cases of those people whom we so frequently meet who go by the name of hypochondriacs. Such folk really do suffer from pains and disturbed sensations, but their sufferings are enhanced and aggravated to a woeful extent by the habit they acquire of looking for them and of expecting them. They, as it were, "hug their misery."

There is yet another point I wish to direct your attention to on the subject of pain. The word is ordinarily associated with uneasy bodily sensations, but I would have you grasp the truth that there is another variety of it which is as distressing and even more grievous to bear with than this. I allude to mental pain—the disquiet, misery, and torture of the organ of thought, the brain. Talk to the average person about anyone suffering from mental pain and he fails utterly to understand what you are speaking about. He quite understands that grief is an unhappy sensation and that joy is a pleasant one, for very few persons have failed in their lifetime to have experience both these states of feeling; but speak of someone suffering from the pain of thought and he fails to grasp your meaning. And yet it is as common a thing in the practice of medicine to meet with and to have to minister to this state as it is to treat ailments of the body which give rise to bodily suffering. The physiologist will perhaps say that I am unnecessarily drawing a distinction between the two forms of suffering, and that they differ only in their seat, both being finally impressed upon the conscious self through the medium of the brain. But there is really a great distinction to be drawn between the two, bodily pain giving rise to distressing sensations, whilst mental pain consists in distressing thoughts. I believe the latter to be the greatest evil of the two, and it is certainly more often fraught with evil results to the sufferer than is the former. It is to be met with in its worst forms in cases of insanity, especially in that variety of unsound mind which is called melancholia. Here the victim is plunged into the greatest misery—a wretchedness, moreover, which is not to be relieved by the use of anodyne drugs, as is the case when physical pain has to be endured. He is also misunderstood by all but the very few who have studied the matter and have thought it out. Nothing is commoner than for the medical man who is attending such a patient to be given advice by outsiders as to his conduct of such a case. He is told to rouse his patient up, and is informed that all that is wanted to effect a cure is that the sufferer shall go more into society and so have pleasant thoughts intruded upon him. You cannot alter the thoughts by any such plan of treatment; in the majority of cases it drives the melancholic person into worse despair than before, and the non-recognition of his state and the unwillingness to treat it in a rational manner is responsible for a great many of the sad suicides we read of, and is also the cause of much confirmed lunacy in the country.

To revert to the distinction which exists between functional and organic disease, from which I have digressed by the discussion I have entered into on the subject of pain, I draw your attention to the point that all organic disease must lead to the disturbance of function in the organs involved by it so that it must perforce be organic and functional at one and the same time, but that functional disease may exist without any change in structure occurring in the organs which are at fault. This is just the difference between the two things. In the organic disease an actual change has taken place in the structure of the parts concerned; in functional disease there is only a derangement of action of the parts concerned. To make my meaning plain I will use as an illustration disease as affecting the heart. A patient is suffering from uneasy sensations at the seat of that organ, and he is rendered miserable by irregular action, and by palpitations at the same part. What is the matter with him? The heart is examined, and is found to be of natural size, in its right situation, and to be free from all sounds which indicate disease of its valves; the patient, let us say, is a great smoker or is a sufferer from some form of indigestion—a malady which frequently disturbs the heart—and on treating these conditions the bad sensations, the palpitations, and the irregular actions disappear. Evidently the function or working of the organ has been disturbed, and we have been dealing with "functional disease." But with a like train of symptoms we find, on examination, the heart to be displaced, or to be larger than it should be, or to yield to our trained ears abnormal sounds when we listen over it. Then we know that it has been altered in structure, and that we are dealing with an "organic disease." The functional disturbance caused by this change in structure may be amenable to treatment, but as it arises from gross changes will be likely to recur from time to time, for, unlike the first complaint, it has a more or less permanent cause.

### Nursing at Netley.

By Our Commissioner.

### A VISIT TO THE CHIEF MILITARY HOSPITAL.

The engine of the train that took me down from Waterloo to Southampton bore on its front a placard announcing "Relief of Ladysmith," so that as we rushed through the country stations people waiting on the platforms had the glad tidings conveyed to them in a novel and unexpected manner. As most people know, the number of visitors to the chief military hospital just now is exceptionally large, and it is a matter for congratulation that the arrangements of the South-Western Company for the convenience of travellers are all that could be desired. As to Netley itself, invalids alighting at that station will soon be able to be lifted from the train and carried straight into their beds. At present they have to be taken in an ambulance up the hill to the hospital, but in a very few weeks this will be altered by the extension of the siding across one end of the grounds. This improvement, it appears, was suggested by the Queen herself, and upon the occasion of her visit on Tuesday she expressed her gratification at the rapid progress it was making. But, as I found almost directly after my arrival, the percentage of patients who are quite unable to help themselves is very limited. When I had been received by Miss Helen Norman, the lady superintendent of the hospital, in her pleasant drawing-room commanding a beautiful view of Southampton Water, we began to talk about the sick and wounded soldiers, and she kindly suggested that before I asked her any further questions I should see some of the sufferers for myself.

### Seeing the Sufferers.

Accordingly, we started on a round of inspection through one of the long corridors which are a great feature at Netley. In the first ward we entered I was struck by the number of men who were taking an afternoon siesta, many of them lying dressed on the outside of their beds.

"Is it compulsory," I asked, "for Tommy Atkins to indulge in a rest at this time of the day?"

"Oh! no," Miss Norman replied, "he generally does it because he likes it. In some cases he needs it, but in others it is a pleasant way of spending an hour."

Most of those who were not dozing were gathered round a huge fire, either reading periodicals or talking. I inquired of some of them whether they felt the cold, and the answer invariably was, "Yes, exceedingly; just as we felt the heat out in South Africa." A point of interest with regard to the men in the wards who still require considerable attention, is that those who had been hit in the thigh invariably complained of feeling pain in the foot. Although it is true that the majority were wounded in the lower part of the body, there were several badly hurt in the head, a considerable proportion had had wonderfully narrow escapes. One poor fellow, who had been severely injured about the jaw, was so far recovered that he was able to converse a little, and a good many were sitting up in bed with their arms in splints or bandages. I asked if Bugler Dunn was still in the hospital, and Miss Norman replied, "He left us a fortnight ago. He is a very nice boy, and was quite unspoilt by all the attentions paid to him."

But there was another interesting lad, suffering from hip disease. He had not come from South Africa, but had been in the same bed for two years. I stopped to speak to him, and with a bright smile he pointed to the quilt on his bed as he said, "The Princess brought me that herself."

Miss Norman explained that when the Princess Victoria of Schleswig-Holstein visited the hospital some time ago she was very much interested in the lad, and that the other day she brought a quilt which she had worked with her own hands. Hearing for whom the gift was intended, Miss Norman was prepared to show her the way to the ward, but he Princess remembered exactly where it was, and walking straight there, had the pleasure of putting it on the bed herself.

### The Dysentery and Typhoid Cases.

After a peep into the operating-room, which is commodious and admirably arranged, and a look at some Röntgen ray photograms, we proceeded to the wards where the sufferers from dysentery and typhoid are treated. The former ward contained about half a score of patients, some of whom looked sadly enough. These men are not able to wait upon themselves, and the orderlies are on duty day and night. The Queen made a special point of giving flowers to the inmates of this ward, and one of them held up his bunch for me to admire. There were only three typhoid patients, one of whom had been suffering from December 6th and is just beginning to recover. He was with the Royal Fusiliers at Estcourt, got ill there with dysentery, and afterwards contracted typhoid. He was able to talk, and assured me that he was now "getting quite well." A young fellow who was taken ill on the ship on his homeward journey had the bloom of health returning to his face. But the third patient, who was sleeping uneasily and had been in danger for five weeks, looked still seriously ill. In the typhoid ward the men said "The Princess" gave the flowers, which were evidently much treasured.

"And now," said Miss Norman, "you have seen all the serious cases. The convalescents are capable of looking after themselves in the wards, in the grounds, or in the library."

But these I had not time to see individually, although they form, I was told, by far the largest proportion of the inmates of the hospital.

### The Great Army Convalescent Home.

"In fact," said Miss Norman, as we returned to her rooms, passing as we went here and there a man apparently practically fit and well, "few of the patients leave South Africa till they have almost recovered. They remain here, however, until they are able to resume duty, and then they get a furlough or go to a convalescent home. But I consider that this is really the great convalescent home for the British army."

"How many patients does it accommodate?" I asked.

"About nine hundred. There are now between five and six hundred men in the hospital, but we shall be full later on, and shall require to use the huts."

"What number will the huts hold?"

"From four to five hundred. They are of German manufacture, and have been purchased through the kindness of the Central Committee of German Red Cross Societies. They are for the use of convalescents only, though they are warm and comfortable. Each will hold ten men."

"We shall have about twelve hundred men home from India soon," continued Miss Norman, "some of whom will come here. The number is exceptionally large, because the sick in India have accumulated. Owing to the war there was no troopship available to bring them back before. But this is the latest time for them. A great many will be convalescent when they arrive."

"I see that the men like to read the magazines?"

"Yes, they appreciate magazines, and they are fond of a game of cards. As a rule they do their own correspondence."

### The Nursing.

"And now," I said, "about the nursing. What is the work of the orderlies?"

"They discharge the duties that usually fall to probationers. We always get a full measure of work out of them. They are very willing and well-behaved."

"At what age are they eligible for admission?"

"Eighteen. Some stay with us for many years, but I like the young orderlies."

"How many Sisters are there now in the hospital?"

"Eleven. The usual number is ten. The Sisters do everything possible for the bad cases, but now that you have seen how little attention most of their patients require you will not share the delusion that they are over-worked."

"Has the Sister who accompanied us round the wards been here long?"

"No. All my usual staff are abroad. The present consists of probationers and Army Nursing Reserve Sisters from different parts of the country, some from general hospitals, and others have been engaged in private nursing. They work very well, indeed. In one sense I have no regular staff. But every probationer has to be here for six months before she becomes a Sister, and sometimes after the period of probation the Sisters stay for two or three years before they are nominated to a vacancy at a station."

"Have the Sisters any responsibility for the diet?"

"They look after the feeding of the bad cases, but the medical officers determine the diet. The diet is very liberal, and every patient has what is ordered him by the doctors, from beef-tea to chicken, and from beer to champagne. All the food and stimulants are supplied by contract."

"What are the hours of duty for the day Sisters?"

"They usually come on duty at a quarter to nine, are off from a quarter to three until a quarter to six, and then on again until half-past eight. There is a good deal of irregular duty, but when the wards are empty they are off for days at a time. The holiday leave is thirty days, and each Sister has also three days four or five times a year. They have a very good time of it on the whole; but when the work is brisk they are expected to do it. I do not care about laggards, but prefer people who can work and people who can play. My opinion is, that, with all these advantages, we get the pick of the nursing world at Netley."

The Pick of the Profession.

And then after learning from Miss Norman of the pleasure afforded to everyone in the hospital by the Royal visit, and especially by the tender sympathy of the Queen—who naturally seemed a little tired after her two hours' tour through the vast building—I made for the station, hearing, however, before I left Netley, warm testimony to the manner in which the lady superintendent fulfils her own responsible duties. Patients and their friends, the Sisters and the orderlies, seem to vie with each other in recognition of her devotion, her skilful management, and her tact. "Right worthily," as one who is well acquainted with her work puts it, "does Miss Norman wear the blue ribbon of the Army Nursing Service."

### Appointments.

Goole Cottage Hospital.—Miss Mary E. Lamb has been appointed Matron. She was trained at Bradford General Infirmary.

The Stephen Cottage Hospital, Dufftown.—Miss E. H. Johnston has been appointed Matron. She was trained at the Western Infirmary, Glasgow, and for over three years was nurse-matron at The Hospital, Lennoxtown, Stirlingshire.

The Nurses' Co-operation Nurses' Home.—Miss Mary Wells has been appointed Matron. She was trained at the Children's Hospital, Pendlebury, and Cumberland Infirmary, Carlisle. Her subsequent appointments have been those of charge nurse at Cumberland Infirmary, sister at the General Hospital, Birmingham, and for the last two years assistant to the superintendent of the Nurses' Co-operation.

### In the City, March 1st, 1900.

From the Point of View of a Nurse in the Crowd.

England knows that every man has done his duty. "Oh! Tommy, Tommy Atkins," you should be a proud and happy man to-day! To-day the throat of this vast metropolis is weary and aching, weary with much shouting of your praises, Tommy! It was a thrilling sight; truly a mad city—mad with enthusiasm, with gratitude, and loyalty.

Amongst the ever-varying crowd one noticed here and there a man in khaki, a nurse or two in uniform, and for a background busy City men, their everlasting money-grubbing forgotten for the time, grave professional men, messenger boys, working men, and all the riff-raff that makes a London mob. "Ladysmith relieved!"—from mouth to mouth it passed, and up, up, as from one mighty throat, rose the shout of thousands! Was ever such a scene?—every 'bus, van, and cart pressed into the service, and all bright in the morning sun with the red, white, and blue of England's flag—not a covered head within a quarter of a mile of the Mansion House—shouts, colour, and laughter everywhere—London's loyal citizens! One felt that for once one was really living, every pulse and every fibre tingling and throbbing, every emotion stirred to its utmost depths. A scene that moved one to tears, and then anon to laughter—that made one thank God for one's nationality and feel how good a thing it is to be an Englishwoman. One thought with glowing pride of the women who have sent their dearest and nearest to the fight ungrudgingly, of the men and women, too, who, for their country's sake, are facing sadness, sickness, and sorrow day by day, with calm and noble hearts and healing hands.

### Novelties for Nurses.

### KHAKI LINEN.

The "Khaki" linen as supplied by Messrs. Harris, of 25, Old Bond Street, is made in two qualities. A thin quality thirty-six inches wide is 2s. a yard; this is more suitable for nurses' dresses, which can be had for 38s. each. They are made with pretty Norfolk blouse, and, if wished, the pleats have tiny pipings of red, blue, or white, which make them very bright and pretty. They wash beautifully, and the wear is most durable. The thicker quality is more suitable for coats and skirts. Nurses who cycle will find the Khaki linen most useful for skirts as it does not show the dust, and looks new again when washed. Besides their new "Khaki" linen Messrs. Harris have many new beautiful art linens in shades of navy, pink, tan, fawn, cream, &c.; indeed, over fifty shades are kept in stock. They will send pattern sheet of all their linens free on application, but we should advise nurses resident in London to call at 25, Old Bond Street, and see them for themselves.

### SINGER'S BICYCLES.

We are informed by Messrs. Singer that they are prepared to supply their excellent bicycles to nurses upon favourable terms. We think many may like to know this fact, so we have pleasure in publishing it.

### A NEW BLOTTING-PAD.

A neat little contrivance is before us in the shape of a blotter and letter weight of a porous white material, which requires no renewing like the blotting-pad formed of paper. The blotter is ornamented with a charming little water-colour sketch, which renders it suitable as a present. It will be found everlasting with ordinary precautions against breakage, and the method of cleansing is simple. It is the speciality of the Patent Everlasting Blotter Company, 8 and 9, Essex Street, Strand, W.C.

Ladies' Bicycles.—The addresses of Anderson, Anderson, and Anderson, Ltd., are 37, Queen Victoria Street, E.C.; and 8, Queen Street, Cardiff.

### The Princess of Wales's Nurses on their Way to South Africa.

THE "KINFAUNS" CASTLE.

One of the Sisters of the Army Nursing Reserve on board the "Kinfauns Castle" sends us the following notes of the voyage:—

This ship is most perfectly fitted up with telephones from one part to another, and the electric light is everywhere. The ventilation is excellent, and the service on board very good; everyone is well looked after, and we are most comfortable. One hundred and fifty tons of coal are burnt daily, and there are forty-two furnaces going; this will give you some idea of the size of the boat. She is like a small town. The bread and pastry are baked by steam, but the big spits on which our joints are roasted are worked by electricity. We have two anchors, each weighing five tons. Huge iron doors can be closed in case of fire. One morning there was fire drill on board. In less than three minutes after the alarm, which comes at any moment without warning, all hands were up on the hurricane deck, with boats ready to lower, &c. The rapidity with which it is all carried out is extraordinary.

MR. KIPLING AND THE HAMS.

Plenty of amusements are provided for the passengers. At the sports some questions were set for ladies. One was, "How many hams have twenty pigs?" My friend, Sister C., at once answered eighty. The judges accepted this reply as correct, consequently there was much controversy. It was even suggested that the butcher should be called! But the judges' decision was final. Rudyard Kipling wrote the following verses to pacify the disconsolate ladies:—

All things were made in seven days
By God the great Designer;
He gave each pig two hams apiece,
Save on a Castle Liner.

Save at "Kinfauns Castle" sports,
As judged by Merrilees,
And then the little squeakers had
As many as you please!

FIVE POUNDS FOR THE HOSPITAL.

Mr. Merrilees won the sweepstakes on the run the other day, and presented the £5 to Sister for the hospital. We were very pleased, as it is sure to be most useful. All the volunteer officers, and most of the men, have been inoculated with the typhoid serum. We were told off by twos to help to prepare and take their temperatures. We were very grateful for even this little bit of work. All have had their turn now, I am glad to say, for the poor fellows' sakes. It would have been much worse for them to have been obliged to lie in their cabins had the weather been as hot as it is now. They have all done well, and were up in 36 hours.

THE CONCERTS AND BALL.

We have also had delightful concerts. One evening Mr. Kipling's "Anchor Song," set to music by Mr. Filson Young, was sung for the first time by Mr. Lewis Thomas. Mr. Young, who is on board, is a war correspondent, and Mr. Thomas a professional, sang splendidly. We all had copies of the words presented to us. The captain, who is an old Scotchman, and one of the best navigators on the Castle Line, has seen 40 years on the service. He is very popular with everyone. He gave us the quaint little song "Mary," which he sang in a very "coy" manner, and so sure was he of an encore that when the vociferous applause was over he produced three other songs, which amused us much. We have had a fancy dress ball, which everyone who took part in it enjoyed immensely. There were the usual collection of cooks, milkmaids, &c.; some of the men dressed as ladies. One man, for whom I trimmed a hat bought at Madeira and decorated it with green and pink crinkly paper as the only thing obtainable, wore a white overall and broad pink sash, lace ruffles, and a fair wig. The hat had strings tied under the chin, and he was much admired. Being about six feet high, I leave you to imagine the effect of this "study after Kate Greenaway." We have some splendid Englishmen on board amongst the first class passengers. Mr. Kipling turned to me the other day during the tug of war, and said, "You could not see a finer or more perfect set of men anywhere; it seems a pity they should have a bullet put through them by the Boers." His two children are dear little things. It was Elsie's birthday yesterday. She wore a wreath of white flowers on her head, and was running about all the morning, saying, "It's not my birthday to-day, it's to-night," because in the evening she was to have a party with a sugar cake, &c. Little John, her brother, is a solid, stolid little man, extremely independent, and the very image of his father.

THE SUNDAY SERVICES.

On Sunday we had an early Celebration at half-past seven a.m. About twenty persons were present. There were if anything more men than women, which showed that many realised they were going to the front to face death. At half-past ten we had service in the saloon conducted by the captain. I never heard anything so impressive as the singing. There were at least three hundred people present and everyone joined heartily, especially the soldiers, in the hymns 391, "Onward, Christian Soldiers," and 376, "O God of Love, O King of Peace."

ARRIVAL AT THE CAPE.

There is one link which binds us all together, the common one of being absolutely without knowledge of where we may have to go when we reach the Cape. Soldiers, civilians, doctors, nurses, are all in the dark as to where duty may call them. As we get nearer the war we realise more what it means to us, for almost everyone is going to take part in it. We arrived at Cape Town twelve hours earlier than we were expected. I never saw anything so lovely as our first view of Table Mountain, with its most perfect tablecloth of white clouds tipped with gold, and the long range of hills lying peacefully under the blue sky of this perfect summer's day. Along the coast lies the town, apparently extending for miles, backed by terrible mountains which appear like slag and make one realise the hardships our men undergo. We are anxiously awaiting orders.

HINTS FOR NURSES.

ANOTHER sister of the Army Nursing Reserve who went out by the same vessel sends a useful list of articles which she thinks a nurse might be glad to consult before purchasing her outfit for South Africa.

Celluloid cuffs and collars, sailor hat, cap, rug, deck chair and cushion, marine soap, opera-glasses, tin of biscuits.

Thick and thin clothing (silk and wool, nun's-veiling), paper collars and cuffs (obtained at Mey's, Newgate Street), bottle and sparklets, tea, condensed milk, tin opener, milk and coffee, marmalade or jam, champagne cork, lantern, holdall for hanging up in cabin or tent, brown boots and shoes (better than black), cholera belts, bath towels. For luggage I found a cabin trunk, brown canvas bag, small handbag, and tin-lined wooden box for the hotel quite sufficient. A basket table bought at Madeira is most useful, as camp furniture is of the scantiest description. Biscuits, chocolate, potted meat, meat lozenges, cake, Bovril or soup powders or tablets, tea basket or extra cups and saucers, tablecloths (for tea), some medicine tabloids, quinine, calomel, &c.; mosquito netting, a few extra medicine glasses and a few glass cloths, ink tabloids, thick shawl and thick golf cape, indiarubber bath, goloshes, mackintosh or even indiarubber boots (very useful if you are in tents). Plain coat and skirt and blouse is useful; sailor hat, white covering for umbrella, thermometers (six), gaiters or spats.

### The Chief Dangers to be Apprehended from Severe Burns and Scalds.

### EXAMINATION QUESTIONS FOR NURSES—RESULT OF FEBRUARY COMPETITION.

The question for the February competition was as follows: "Define the different degrees of danger to life from various kinds of severe burns and scalds, and the means you would take to obviate fatal results."

### The First Prize.

"Nurse Isabel" again takes the first prize, her thoughtful, practical, and lucidly expressed answer being far before those of all other competitors. This continued success will disable her from taking a first prize again until May, 1901. Those who have hitherto failed will do well to ask themselves why they have done so. There are several causes. One is carelessness; this is the cause of more downfalls than any other. Another potent cause of failure is a desire to show off. I will not quote names, but one paper has been sent in so stuffed with medical terms that the candidate, having spent her mental energies upon those, has failed in noting some most important points. There is a further cause of failure that I should like to mention, and I hope that nurses may lay the subject to heart. Even so short a time as ten years back instruction given to probationers in hospital was somewhat meagre; it is true that there were good lectures given in some hospitals, such as the "London," but the practical instruction given by sisters of wards was, as a rule, somewhat scanty. The result of that was to cut both ways; lazy and unenthusiastic nurses learnt but little, did what they were told more or less well, and were content to "muddle along" somehow. The "born nurse" did otherwise, as but little help was given her. She was compelled to find the thorny path of knowledge for herself, and what cost her so much to acquire was not easily forgotten. Now all is different; sisters are compelled to give instruction, and, on the whole, the system is better, but it has its disadvantages like all human systems—it does not compel nurses to exert their brains. They have learnt their profession to a great degree like parrots, and if a question is put in a different way to what they expect or are accustomed to they are all at sea; they do not understand the practical questions involved, and answer like a handbook.

### The Second Prize.

"Nurse Edith" comes out second. Her answer is short, but it shows that she understands what she is writing about. She touches the very kernel of the question in saying that "the severity of the shock would be according to the extent rather than the depth of the injury." It was to arrive at that fact that the setter of the papers gave the question.

### No Honourable Mention this Time.

The cards awarding honourable mention are ready, but this month no candidates besides the prize winners are considered good enough to receive them. It is painful but necessary to mention that no less than five papers were sent in that were copied almost verbatim from nursing handbooks. These were at once consigned to the wastepaper basket. Nothing could be more dishonourable; we rely upon the honour of candidates not to use such books, and they fall lamentably short of our trust in them. It is a discouraging thought, that women who will betray trust in little things are only too likely to do so in those of a serious character. Honesty and fidelity of purpose should be the distinguishing characteristics of a nurse.

### Nurse Isabel's Answer.

The different degrees of danger to life arising from various kinds of severe burns and scalds correspond directly to the extent of burnt surface, the position of the injury, the depth of tissue destroyed. That is to say, shock will be occasioned by a severe burn on whatever part of the body it occurs, particularly in the case of a child, but the degree of this resultant shock will be in direct correspondence to the extent of the surface injury, and it will be yet more marked if the injury is to the chest or abdomen. If, in addition to these important features of extent and position, the tissue is deeply destroyed, we have a type of the burn most dangerous to life. The presence or absence therefore of these three features, and their degrees of prominence, determine the degrees of danger arising from the injury. Scalds or burns of the mouth and throat must also be classed among those most dangerous, as the risk of suffocation is great, and will demand the utmost watchfulness and preparedness for tracheotomy on the part of the nurse. In the case of burns of the head and face, erysipelas is a complication to be feared and guarded against. In addition to the immediate danger occurring from shock, the nurse has to bear in mind the congested internal condition likely to follow from the grave depression of the circulation. Bronchitis, pneumonia, brain and kidney trouble may all supervene, and must be guarded against as far as possible. To obviate fatal results, therefore (and the most dangerous hours are those mmediately following the injury), she must, in the first place, protect the part forthwith from air until the proper dressing is ready; in the second, attend carefully to the condition of shock. The patient must be laid on a bed between blankets with the head low; hot bottles must be placed at a little distance from the feet (in more serious cases between the thighs, and near the sides also) and a stimulant administered. Pulse and respiration must be watched. Warmth, rest, and quietness are essential. Brandy and sal volatile in small repeated doses, or ether and ammonia, are all useful in serious adult cases. With a child small quantities of warm milk and brandy will be best, and attention should be paid to the proper passing of water. If the patient cannot swallow an enema of brandy and beef-tea may be given. It must be borne in mind that even when the patient appears to have rallied relapse is very liable to recur; therefore watch must be maintained on the pulse and general indications. On the other hand the stimulant treatment must be carefully moderated as the patient really revives. When the first shock has passed off the next danger arises from the congestion before mentioned, which is now likely to follow. The nursing of the case must therefore be continued for another fortnight at least, with every precaution against chill and exertion.

### Nurse Edith's Answer.

The first great danger to life from a severe burn or scald is from shock, the severity of which would be according to the extent rather than the deepness of the injury; the precautions taken by the nurse being to wrap the patient in warm blankets, give him hot bottles, and stimulants.

Secondly, fever, and in deep burns it would be the nurse's duty to watch for symptoms of injury to the internal organs, especially ulceration of the duodenum, when the stools sometimes contain blood. She would then give light diet, no stimulants, and be very careful there should be no exposure during the dressing of the burns, a small part only being done at a time.

Thirdly, exhaustion from the great amount of suppuration taking place over a large surface, the patient requiring nourishing diet and stimulants.

Fourthly, kidney disease would have to be watched for, the action of the skin having been stopped, when milk diet could be given until receiving directions from the doctor. In all cases the patient to be kept warm and free from any exposure.

### Question for March.

"What do you consider the most important points to be attended to in the nursing of typhoid fever?"

### Nursing at Wynberg,

THE PRINCIPAL CAMP HOSPITAL.

One of the Irish Nurses at No. 1 Hospital writes under date of January 22nd to Mrs. Treacy, superintendent of the City of Dublin Nursing Institution, as follows:—

"We arrived here safe and sound after a fine knocking about the last days of our journey. Up to the present we are together, but we may at any moment be sent off to different places.

"This is the principal camp hospital; it holds over 1,000 patients, 21 nurses, medical staff, &c. We are very comfortable, and the army people have a house furnished (£18 per month) for the nurses. Each four nurses are entitled to a servant. We have a regular 'Topsy,' and I am appointed housekeeper for the time being. We are allowed servants, wages, coal, and £1 per week each for food extra; but the price of food is awful, and it will take us all our time to manage it from above amount. The patients are six in a tent, and so many tents for each nurse. We have the officers' quarters. Patients are coming down every day from the front.

"There is no system whatever as to work. You can do as you like. The soldiers are very nice and would do anything for us. The orderly who met us at the train was a Munster Fusilier. The patients give interesting accounts of the battles, and are anxious to get back to the fight, especially the reserve. They deserve all the people at home are doing for them and more. One man told me after the last battle, after fighting twelve hours on a biscuit and a drink of tea, they had to drink water out of a place there were 150 Boers lying dead in.

"A sister of a patient of ours called on us, and many of the leading ladies of the locality as well. Everyone is very kind to us, and the orderlies are civil and capable.

The Queen's Chocolate.

"The men have just received the chocolate from the Queen, and the moment they saw us they had the boxes out to show us. They are sending them home by registered post to their families for safety. They are little oblong gilt boxes with the Queen's photo and autograph. They appear to think more of them than of a Victoria Cross. Our nurses' medals are greatly admired. One colonel especially informed the other nurses on board the 'Moor' that 'they might get the Victoria Cross, but that they would not bring back that Order' (St. John of Jerusalem in England).

"The orderlies have to crawl on their chests amidst the firing, and draw the wounded and dead on stretchers off the battlefield.

Cholera Belts for the Nurses.

"Wynberg is a most exquisite suburb of Cape Town, in a forest of pines and oaks—and not too warm. Cape Town we did not like; it was too hot and dusty. We were vaccinated before leaving the ship, and have now to get cholera belts, as several have got dysentery. One nurse died. We are all three well up to the present, and happy.

"The view of Table Mountain here is exquisite. Our house is surrounded by lovely tropical plants, and the house is furnished almost luxuriously. We think the mosquito nets funny over our beds, and the singing of frogs outside our windows at night, like crickets. Our servant is very capable, cleanly, and very black.

"The nurses get drenched going round the tents sometimes, as the rain comes straight down, but this is the dry season. We hope to have heard from you before this is posted, and love and regards from each of us to yourself and all the nurses. We have a photo in our dining-room which was taken at the Viceregal party. I brought it with me."

### English Nurses in Boer Hospitals.

By an English Nurse.

Trained nurses in South Africa avoid, as they might the plague, entering a hospital governed by a Boer committee and under Boer control. Dutchmen do not realise that white nurses cannot do as much as Kaffirs, not that Kaffirs do work hard, but they are expected to. The white nurses are also expected to, but they do it. Before the war there were several English nurses in the Bloemfontein and Pietermaritzburg hospitals. All of them say that they had a terribly hard time, and that wherever Boers have any power the nurses are treated very badly. An English nurse as a rule only accepts service in a Boer hospital as a matter of "Hobson's choice," just while waiting for something better, or when private nursing becomes slack.

"In Boer hospitals the women nurses are left at night entirely at the mercy of the natives, even with delirium tremens and insane cases. It is not a Boer custom to show consideration for women, and when an English nurse suggests that a watchman or night porter should be within call, she is taunted by a Boer committee as "a weak-stomached Britisher." The slightest cut on a Kaffir's head makes him delirious and homicidal. Matabele boys, on the contrary, who are kept under strict prohibition laws, may have a bullet through the skull, but, unless it reaches the brain, they rarely suffer from delirium. Wounds heal wonderfully in South African air, and so would those of the Kaffirs were meat and strong drinks kept out of the Dutch hospitals. Sometimes a good-natured Boer official in a hospital will allow an English nurse to have a coolie with her at night in a male ward, but coolies are timid and no match for Kaffirs. Consequently, the nurses in the Dutch hospitals often have dramatic experiences during the night with delirious natives making homicidal attacks on fellow-patients.

A nurse recently on duty at the Bloemfontein Hospital says she thinks her nerves will never recover from the shock of being alone on four consecutive nights in a ward where a homicidal Kaffir was making continued attempts to strangle the patient in the next bed. It was very exciting, but terribly wearying. One night he escaped, but was brought back on the following day by the police, who said that they could neither control nor accommodate him. So he was handed back to the care of the terrified night nurse. On the fifth day the brain symptoms subsided; but his nurse declares that the memory of what she endured on those four awful nights will never subside. The hardy stalwart Boer woman does not mind experiences of this kind. Indeed, as a rule, she is so physically strong that she can deal almost as well as a man with a Kaffir made rabid by strong drink. Native patients frequently strike the nurses, or anybody else who "interferes" with their views of medicines, bandages, and other things medical. Boer clergymen visiting the wards often hit the patients back again, and nobody is surprised.

Boer hospital committees complain that "English nurses are so restless and hard to please." Considering the conditions under which they have to work in the Dutch-managed institutions, it is not surprising that they "flit" frequently. At Bloemfontein and Pietermaritzburg hospitals the new nurses come on a month's trial. At the end of this probation they are free to give a month's notice, and English women usually take advantage—gladly, gleefully—of this privilege.

No European nurse who has served in a Boer hospital can view with anything but sheer delight the near prospect of having all the institutions of South Africa under sound, wholesome British rule.

### Echoes from the Outside World.

AN OPEN LETTER TO A HOSPITAL NURSE.

How desperately disappointed the people of Bordighera will be at the Queen's decision not to visit their picturesque town this spring! I think that even by us, her subjects, the announcement that she will not leave England has been heard with regret rather than with rejoicing, because we feel how much the dear lady is giving up for our sakes, and tremble lest she be relinquishing a change which is almost imperative in the interests of health. For many years she has not had such a severe strain put upon her strength as during the sad months which have elapsed since last October, and yet she has never given way. Scarcely a repulse has been recorded but the Queen's telegram has brought sympathy and encouragement; not a success has been scored without a few congratulatory lines from Her Majesty; the wives of the officers who have been left desolate have had touching letters addressed to them, with requests in many cases for photographs; and even the little bugler boy, who distinguished himself, has not been forgotten. All this means a very close following of the daily events of the war, with much thought and care that all should be duly remembered. Added to this, after tiresome journeys there have been long hours spent in visiting and talking to the wounded soldiers sent home to hospital, with the consequent fatigue and exhaustion which must inevitably follow after much trying exertion when one is no longer young. Now, as a culminating point, comes the voluntary sacrifice of sunny skies, bright surroundings, and pleasant holidays, and the resolution not at the present juncture to leave the land which, although it be the one over which she reigns, is always particularly cheerless and disagreeable during March and April. The French should be especially pleased, because, if she does not pay "la belle France" a visit as usual this year, she is anyhow not giving the preference to any other country.

---

It is not only humble people like ourselves who cannot always do as we will with our own. There was something pathetic in the statement made the other day by the Prince of Wales at the opening of the new workmen's dwellings erected by the London County Council in Shoreditch, that though he was "the nominal owner of some property in Lambeth, he had unfortunately no control over it, though the public thought it his own." To know that, though families were living in houses both insanitary and ill built, and paying rent for accommodation hardly fit for animals, nothing could be done to remedy matters, must be intensely galling to such a good landlord as the Prince of Wales has proved himself to be at Sandringham. But the property has been granted to its present holders on a lease, and as yet there is no Act of Parliament which will help the real landlord to deal summarily with the leaseholder who allows his property to become insanitary. Therefore, until 1909, when the Lambeth houses once more return to their original owner, the Prince will have to try and rest content with things as they are. Afterwards a better state of things may be hoped for, for His Royal Highness promises himself the pleasure, to use his own words, "of seeing the tenants on my Lambeth property as happy, as comfortable, and as well cared for as I am able to say they are in Norfolk." It is utterances like these, pregnant with promises, which make the Prince the popular son of a popular mother.

---

Do you think any of us will ever forget March 1st, 1900? The scene in the City was wonderful, but I will not touch upon that, because you will find a graphic account written by one of yourselves in another part of the paper. Even in the suburbs the joy and gladness was so infectious that it touched all, young and old, rich and poor alike. Everyone who had a flag or a piece of bunting left from the Jubilee, hunted it up and hung it out at once; those who had no stores put away rushed to the shops, and sewing machines and fingers flew apace; even the very dustmen managed to secure flags, and mounted them over their unsavoury carts in much glee. The smiles on the faces of the people were good to see; the children went about humming "God Save the Queen," a reminiscence from the vocal outburst of loyalty which occurred during the morning in nearly every school, and the very babies lisped of "Adysmiff," having learnt it from parents or nurses' lips. It was delightful to feel so overwhelmingly joyous and thankful. And if we, who had only looked on, felt like this, what must the feelings of the sufferers inside Ladysmith have been? The assertion of an eye witness, "that passing down the street was like passing through the wards of a fever hospital, so yellow and hollow were the faces of the besieged, the skin drawn tightly over the cheekbones, and the uniform hanging in wrinkles over the gaunt forms," helped one a little to grasp the state of affairs. That the distress and scarcity were much worse than the brave defenders ever allowed us to know is proved by the latest prices paid before the relief came. A pot of jam cost 31s., a fowl 18s. 6d., a dozen potatoes 19s., eggs 4s. each, a 14 lb. tin of oatmeal 60s., whilst those who managed to secure some violet powder to make a blancmange were lucky indeed. I fear, too, from the latest news that Mafeking is now experiencing the same pinch. A soup kitchen has been opened with the object of converting horses, stray dogs, and the heads and feet of oxen into human food. But relief for Mafeking will probably come as unexpectedly as it did to Kimberley; and elsewhere all goes well. General Brabant has driven the Boers off from Dordrecht, and General Gatacre has occupied Stormberg, the Boers having evacuated it. So that at last we can say Natal is clear of Boers.

---

Unfortunately, nurses as a general rule are not affected by any addition made to the Income tax, because their salaries are usually far below £160, the limit of exemption. Therefore the addition of 4d. in the pound which the Chancellor of the Exchequer considers it necessary to impose will not disturb your equanimity. In fact, the only feature of the Budget which you are likely to feel is the impost of 2d. per lb. on tea, and even that is scarcely likely to cause you to make yourselves even one cup of tea the less during the year. But apart from personal considerations, it is interesting to note that Sir Michael Hicks Beach has done his best to make both the masses and the classes pay for the war. Thus, there is 1s. a barrel of 36 gallons on beer, 6d. a gallon on spirits, 4d. a pound on tobacco, and 6d. a pound on foreign cigars. One wonders that sugar, which is so ridiculously cheap, and is so much wasted, was not selected; and an export duty of 5s. per ton on all coal purchased in the United Kingdom for use in foreign countries would, in the opinion of some of the wisest judges, have been statesmanlike policy. The increased taxation does not, however, provide anything like half the amount required to defray the cost of the campaign. No less a sum than thirty-five millions has to be borrowed at once, and about eight millions later on. In short, the war forced upon us by President Kruger has already cost us just sixty millions, or more than half as much as the total revenue of the country Just think what might have been done with a sixth of that sum for the good of the hospitals!

### The Matron's Corner.

PERSONAL INFLUENCE.

"HE who serves by being does the best service." Yes, when all is said, it is you yourself, your life, your character, your personal influence which affect the hospital you rule—for good or ill. And just as the life of a Court sets the tone of the life of a country, so does a matron's character and influence set the tone of a hospital. It is by what you are, not by what you say, that your subordinates are led.

It is much easier to preach than to practice—like the famous preacher who said to his congregation, "Do as I say, and not as I do!" It is easy to imagine how many of that ongregation obeyed his maxim.

To be a good matron involves the same quality as is involved in being a good leader. That quality is single-mindedness.

Why did Lord Kitchener's campaign in Egypt succeed in such marvellous fashion? Because of his perfect single-mindedness. He had one aim; everything he did, every step he planned, to the minutest detail, led up to that one aim. And he imbued his subordinates with the same spirit. One of the newspaper correspondents noted that wherever the Sirdar was every soul worked his very utmost, impelled by that one man's impelling energy.

Not that Lord Kitchener ever said much. He is not a man of words, but those under him were influenced by the strength of his aim to make it theirs too, and to carry it out in its entirety.

In the same way, it is the woman of few words and of single-minded aim who sways her subordinates best. I take it for granted that your aim is to bring your hospital up to the highest ideal, and it is in following that with a single mind that your personal influence will tell.

> "He did too many grandnesses to note
> Much of the meaner things about his path,
> And stepping there, his face towards the sun,
> Stooped seldom to pluck weeds or ask their names."

The words fit our conception of a great ruler. "Stepping there, his face towards the sun," is what one expects of a person too great in soul to stoop to littlenesses, and to what is evil and low—like weeds in his path; but whose face, uplifted in the sunlight, lifts others' faces to the sunlight too. There is a saying that each of us influences for good or evil every human being whose life touches ours. If this be true of each individual, how much truer must it be of one in any way set above his fellows? And yet, after all, the greatest influence of all is the most unconscious. It does not do to go round, so to speak, with the idea of influencing our fellows. "It is they who are best who do best"; but, as matrons, you have to face the fact that your personal influence is enormous, and it cannot be a neutral matter—you influence either for good or for evil.

When I began to think of these articles these beautiful words of George Eliot came into my mind as a strangely-fitting motto for a matron's corner, and I still think that they suit it as perhaps no others would:—

> "May I reach
> That purest heaven, be to other souls
> The cup of strength in some great agony,
> Enkindle generous ardour, feed pure love,
> Beget the smiles that have no cruelty,
> Be the sweet presence of a good diffused."

### Miss Dashwood's "At Home."

MISS DASHWOOD, honorary secretary to the Nurses' Union, gave her annual "At Home" to members and friends on Thursday, March 1st, at her residence, 39, Bryanston Square, About two hundred took advantage of her kind hospitality. Indoor uniform, varying with the social status of the profession, was the order of the day. Tea was served in the spacious dining-room, but those who had to leave early and those who came late were also well provided for. Miss Cornwall, L.R.C.P.S. Edinburgh, from the Duchess of Teck's Hospital, Patna, gave a most interesting account of her medical missionary work. She described the difficulties, and mentioned that she had often been called out to see a patient requiring immediate operation, but on arriving, instead of being allowed to set to work, had been requested to sit down and be fanned while the family counted their beads (like rosaries), "Yes," "No," "Yes," "No," and according to what the last one was the operation was done or not. Dr. H. Lankester, of the C.M.S., spoke on Medical Missions in India, and several recitations and singing followed. Before parting, with the relief of Ladysmith in mind, the Doxology was sung.

### Presentations.

CITY OF LONDON HOSPITAL FOR DISEASES OF THE CHEST, VICTORIA PARK.—Mr. Joseph Benson, the chairman of this hospital, entertained the patients to tea last Friday, and afterwards lantern views in connection with the war in South Africa were shown. Later in the evening the nurses were also entertained by Mr. Benson, and during the evening a small presentation was made on behalf of the committee of management to the matron, Miss Beatrice Jones, who has since sailed for South Africa to nurse the sick and wounded soldiers. A similar presentation was also made on behalf of the staff of the hospital, symbolising the esteem in which Miss Jones is held by all who are associated with her in the institution.

THE IRISH BRANCH OF QUEEN VICTORIA'S JUBILEE INSTITUTE FOR NURSES.—Miss Dunn, the late general superintendent of the Irish Branch of Queen Victoria's Jubilee Institute for Nurses, having recently resigned her post, has been presented by the Council of the Queen's Institute with a very handsome solid silver tea kettle, bearing the following inscription: "Presented by the Council of Queen Victoria's Jubilee Institute for Nurses to Mary L E. Dunn, Superintendent of the Irish Branch from June 1st, 1890, to December 25th, 1899, on her resignation, and in recognition of her success in organising the work of the Institute in Ireland."

### To Nurses.

WE invite contributions from any of our readers, and shall be glad to pay for "Notes on News from the Nursing World," or for articles describing nursing experiences, or dealing with any nursing question from an original point of view. The minimum payment for contributions is 5s., but we welcome interesting contributions of a column, or a page, in length. It may be added that notices of entertainments, presentations, and deaths are not paid for, but, of course, we are always glad to receive them. All rejected manuscripts are returned in due course, and all payments for manuscripts used are made as early as possible at the beginning of each quarter.

### Everybody's Opinion.

[Correspondence on all subjects is invited, but we cannot in any way be responsible for the opinions expressed by our correspondents. No communication can be entertained if the name and address of the correspondent is not given, as a guarantee of good faith but not necessarily for publication, or unless one side of the paper only is written on.]

### ST. GEORGE'S INFIRMARY, FULHAM ROAD.

"ONE OF THE STAFF" writes: I wish to ask your opinion of the subjoined "form." The staff of the above infirmary have each received one from the clerk to the Guardians, and I think I may say they one and all object to sign it. Do you think it is right for the Guardians to ask us to do so? Certainly, it appears to me to be unusual to be asked to submit oneself to a "medical examination" by any doctor whom the Guardians may appoint for that purpose at any time they may wish to do so. I myself consider it a great insult, and should certainly object to a report being made to them of any little ailment I might be suffering from. It might possibly get into the *Westminster and Pimlico News*, and as all the patients in this infirmary see that newspaper it would be most objectionable. There is no reason given why the Guardians have made this resolution. Is it legal, is it customary in other institutions, and do you think we are justified in refusing to sign the "form"? The Guardians appear to think they can do just as they like with those in their service, and it is time that we offered some protest. Nurses who work hard, owing to the small staff employed at this infirmary, for twelve and a-half hours a day are quite likely to get sick occasionally, but this does not warrant the Guardians requiring us to consent to a medical examination at any time they may wish to do so.

The following is the form referred to: St. George's Union, London. I do hereby consent to my being transferred to a hospital should I contract any infectious disease whilst in the service of the Guardians unless I am at once removed from the ——— by my friends. I do also consent to submit myself to a medical examination by any doctor whom the Guardians may appoint for that purpose at any time they may require me so to do, and I consent to his reporting to the Guardians the nature of the ailment from which I may be suffering.

### A COMPLAINT FROM NATAL.

"NURSE ANNETTE" writes from Durban: I should esteem it a great favour if you would grant me a little space in your valuable paper in order that some grievances under which nurses—refugees from the Transvaal—are suffering either from the hands of the military or some other authority may be made public. I will simply state my own case as it is typical of hundreds of others. In July last I wrote to the Base Hospital, Maritzburg, offering my services, as I was and am most anxious to help the absent-minded beggars who so nobly and as one man have come to help us in our sore need. I am a thoroughly trained nurse. I have had a large experience in all branches of nursing. I have held the post of hospital matron for some time. In reply to my letter of July, I on August 14th received a letter asking for copies of testimonials, which request was duly complied with. On August 19th I received a letter informing me that my name had been duly entered on the register for use in case of complications arising. A few days later my papers were returned by the colonel in command of A.M.S.C., stating in case hostilities commenced my services were accepted. I waited for weeks expecting a summons daily. War was declared, I wrote again reminding the authorities that my services were still at their disposal. To this I had no reply. On January 10th I obtained a permit to see the P.M.O. I laid my case before him, informing him at the same time that I am thoroughly seasoned to the climate, am a good surgical nurse, and understand enteric and dysentery, as perhaps few English nurses do. I was offered a subordinate position on one of the hospital ships at Durban, which I declined. A few days later another offer of nursing in the Assembly Hospital, Maritzburg, I also declined, as I consider my experience and abilities are looking for superior employment to that of a mere probationer. The only apparent objection to my employment was that I was a civilian nurse. I should very much like to know why we who are on the spot should be treated in such a way, when all the time nurses are being imported from everywhere.

### DANCES IN HOSPITALS.

"NURSE ETHEL" writes: I do admire the matron who wrote three weeks back against dances held in our hospitals. I consider them utterly out of place. The hospital was built to admit suffering humanity, and not for dancing. Surely if nurses desire to go to a dance they can by special permission from the matron do so, for numerous are the number of them held in our large cities and towns. I think we should put ourselves in our patients' place, and consider for one moment how we should like noisy dancing music played in sound, more often than not, of the wards containing our suffering brothers and sisters. In one only of the hospitals in which I have nursed was a dance given annually to the nurses, and several days before the dance came off, the minds of some of the nurses were almost entirely given up to the coming pleasure; while the laughing and talking in the wards, and the choosing of partners amongst the nurses and students in the presence of the patients was anything but becoming in the wards. For that little season patients who should be our first consideration were more often neglected. It is folly to say that such dances do no harm. In many cases they do more harm than good. God has committed into the hands of nurses a great and noble work. Let us see to it that we are whole-hearted in the sphere in which we are called. Then only shall we succeed and be real comforters to sufferers. I am looking forward joyfully to the day when dances in our hospitals will be a thing of the past. It is good to be friendly to an extent, but over-familiarity breeds contempt in the medical profession.

### Minor Appointments.

MAIDSTONE WORKHOUSE.—Miss Perrott has been appointed Charge Nurse. She was trained at Bracebridge, Lincoln, and has since been nurse at Tunbridge Union.

ST. MARK'S HOSPITAL FOR FISTULA.—Miss Louie Forrest has been appointed Sister. She was trained for four years at St. Mary's Hospital, and has since been for a year at the Cancer Hospital, Fulham.

CHESTERFIELD HOSPITAL.—Miss Helen Mapson has been appointed Charge Nurse. She was trained at Grimsby Hospital, and has since been sister at North Staffordshire Infirmary and sister at the Royal Infirmary, Derby.

CHILDREN'S HOSPITAL, PITSMOOR, SHEFFIELD. — Miss Emily Berry has been appointed Charge Nurse. She was trained for three years at the Fir Vale Infirmary, Sheffield; also two years at the Fever Hospital, Cleckheaton.

DUDLEY UNION INFIRMARY.—Miss Marie M. B. Newbury has been appointed Superintendent Nurse. She was trained at the Royal Albert Hospital, Devonport, and has since been superintendent nurse at St. Mary's Infirmary, Islington.

PORTSEA WORKHOUSE INFIRMARY.—Miss Amelia L. Johnson and Miss Nellie Stewart have been appointed Charge Nurses. Miss Johnson was trained at Poplar and Stepney Sick Asylum, and has since been engaged in private nursing. Miss Stewart was trained at the Eastern Fever Hospital and St. Pancras Infirmary.

SWINDON ISOLATION HOSPITAL.—Miss Mary Garland has been appointed Charge Nurse. She was trained at the Devon and Exeter Hospital. Her subsequent appointments have been staff nurse at the North Devon Infirmary for one and a half years, staff nurse at Stafford Infirmary for one year, and private nursing for the last six years at Worthing.

MANCHESTER CHILDREN'S HOSPITAL, PENDLEBURY.—Miss Helen B. Makin has been appointed Night Sister. She was trained for three years at Leeds General Infirmary, and has since been ward sister and night sister at the same institution. She has also been sister of the children's wards at the Royal Infirmary, Wigan, for two years and four months.

ROYAL BERKS HOSPITAL.—Miss Elizabeth Davies has been appointed Sister. She was trained at Swansea General Hospital, and has since been sister at Rotherham Hospital for two years, and sister at the Royal Infirmary, Aberdeen, for three and a half years.—Miss S. Macaulay has been appointed Sister. She was trained at Cardiff Infirmary, and has since been sister at Bristol Hospital for Children and Women for three and a half years.

### For Reading to the Sick.

Be careful for nothing, but in everything by prayer and supplication with thanksgiving let your requests be made known unto God.—*Phil.* iv. 6.

Be not afraid to pray!—to pray is right—

Pray (if thou canst) with hope; but ever pray,

Though hope be weak, or sick with long delay!

Pray in the darkness, if there be no light!

Far is the time, remote from human sight,

When war and discord on the earth shall cease;

Yet every prayer for universal peace

Avails the blessed time to expedite!

Whate'er is good to wish, ask that of Heaven,

Though it be what thou canst not hope to see:

Pray to be perfect, though material leaven

Forbid the spirit so on earth to be:

But if for any wish thou dar'st not pray,

Then pray to God to cast that wish away.

—*H. Coleridge.*

True devotion does not depend upon feeling.

**Reading.**

We must strive to be filled with a consciousness of God's presence everywhere and in everything; to realise that He is truly present in His immensity as we are actually present to ourselves. If once anyone can confirm himself in this habitual sense of the presence of God the shafts of the Evil One will be powerless to harm him. It is our levity and our interest in earthly things which surround us, that hinder our realising it. We cannot keep up a perpetual thought of God, but renewing it by a momentary glance at each thing we do will result in a constant and lively sense of His presence. The very essence of Christian life, the mainspring of it, is a continual reference of the soul to God. Each uplifting of the heart, each ejaculation as it darts upwards, touches the heart of our dear Lord, and brings a fresh supply of His Grace to us. . . . If we believe God is always at hand, always ready to hear, we should take delight in telling Him of our pains, cares, woes, and hopes as they flit by. . . . A momentary uplifting of the heart to God, "without Thee I can do nothing;" I am Thine, save me, or the like, will help us. And He who thinks much of God will daily think less of Himself.—*Sidney Lear.*

Those who are weary with a long and tedious illness cannot often pray; but God is not unjust; He knoweth our desires. Thou who liest fretting there, and canst not raise thy thoughts, thy voice in definite prayer, thy still desires, thy silent longings, have a voice of deepest pathos and compelling power in thy Father's ears. . . . Do not despair, however faint thy voice, or weak thy thought, however short thy prayers, thy wish to pray may sometimes be the very prayer itself.—*E. A. D.*

A prayer in a hour of pain,
Begun in an undertone,
Then lowered, as it would fain
Be heard by the heart alone.
A throb when the soul is entered
By a light that is lit above,
Where the God of Nature has centered
The Beauty of Love!
The world is wide, these things are small,
They may be nothing, but, they are All.

—*Houghton.*

### Where to Go.

Grosvenor House.—Thursday, March 15th, half-past three. Annual meeting of Invalid Children's Aid Association.

### Notes and Queries.

The Editor is always willing to answer in this column, without any fee, all reasonable questions, as soon as possible.

But the following rules must be carefully observed:—

1. Every communication must be accompanied by the name and address of the writer.
2. The question must always bear upon nursing, directly or indirectly.

If an answer is required by letter a fee of half-a-crown must be enclosed with the note containing the inquiry.

*Supplementary Training.*

(222) Having been trained in India at a large European general hospital (150 beds), I am desirous of obtaining some extra training (surgical chiefly) in a large hospital, provincial preferred. How could I do so? By paying? or would it be possible to obtain the knowledge for services only? I hold a three years' certificate from my training hospital and also one for monthly nursing, and I have been doing private work for a European hospital for the last three years. I am an English lady by birth, and I wish to have some insight into English hospital work, as I do not want to remain always out here.—*India.*

With your qualifications you ought to be able to obtain paid work in an English hospital. When you come to England why not insert a somewhat full advertisement of your requirements and qualifications in the "Mirror"? This seems the most direct method of obtaining your wishes.

*Medico-Psychological Exam.*

(223) Could you inform me if it is possible to go in for the Medico-Psychological Exam. in May, without belonging to an institution. I have have had the requisite amount of training in an asylum, but I am at present with a private case.—*E. M. V.*

Write to the Registrar, the Medico-Psychological Association, 11, Chandos Street, Cavendish Square, W., for particulars.

*Maternity Nursing.*

(224) Kindly let me know the law in such a case as the following: A lady engaged a maternity nurse in November for April. She has now written cancelling the engagement, as she has met another nurse whom she likes better. Can the nurse who was first engaged (and who refused two cases in consequence) claim full fees, anything in lieu of board, &c., or compensation?—*Nurse Ruth.*

The case is evidently one of breach of contract, and Nurse Ruth would do well to consult a respectable solicitor; that is, of course, if she has proof of her statements either in writing or by witnesses.

*Dispensing.*

(225) My sister and I have a great wish to become dispensers. Would you be kind enough to advise us whether there is any opening for the same, and if so, should we have to train at a college; or if we could enter a hospital as probationer dispensers?—*Doris B.*

There is said to be a good opening for well qualified women; but the training of such is long and expensive. Apply to the Secretary, Pharmaceutical Society, 17, Bloomsbury Square, W.C., for full particulars. The Secretary, the Women's Medical School, Handell Street, W.C., can sometimes give the names of lady chemists who take pupils.

*Abroad.*

(226) A friend and I are very anxious to go out to one of the colonies as private nurses to work on our own account. We should take money and good testimonials with us. Would you kindly inform me the best places to go to? 2. Do you think Chicago, New York, or New Zealand would be good?—*Colonies.*

You could not do better than write to the Secretary, the Colonial Nursing Association, Imperial Institute, S.W., for information. (2) The United States is not a happy field for the enterprise of English nurses, whilst New Zealand has plenty of home-trained nurses.

*Army Nursing.*

(227) I write to you as I wish for information regarding the new staff of nurses for the daughters of gentlewomen that is being formed for the army. I want to know how to enter, age, &c. I presume you publish all answers in your paper The Hospital, so I enclose 2d. for you to send me the copy in which you give me your answer, and 1d. for postage.—*L.P.*

You do not say to what staff you refer. Possibly you may mean the Army Nursing Service Reserve. If so, the hon. secretary, 18, Victoria Street, will forward particulars on application.

*Flat Foot.*

(228) May I ask if you can give me any information about flat foot? Can it be cured? What is the usual treatment? Does one ever lose the use of a foot through it?—*M. S. F.*

In the "news" columns of The Hospital "Nursing Mirror" for June 10th and July 1st you will find two paragraphs telling you all you wish to know. The first is headed "Skipping as an Exercise for Nurses" and the second "Flat Feet." Ten minutes' tip-toe exercise each day you will probably find of great benefit.

*Standard Books of Reference.*

"The Nursing Profession: How and Where to Train." 2s. net.
"The Nurses' Dictionary of Medical Terms." 2s. 6d. net.
"Burdett's Series of Nursing Text-Books." 1s. each.
"A Handbook for Nurses." (Illustrated.) 5s.
"Nursing: Its Theory and Practice." New Edition. 3s. 6d.
"Helps in Sickness and to Health." Fifteenth Thousand. 5s.
All these are published by The Scientific Press, Ltd., and may be obtained through any bookseller or direct from the publishers, 28 & 29, Southampton Street, London, W.C.

### Travel Notes.

By Our Travelling Correspondent.

### XLVI.—THE PASSION PLAY AT OBERAMMERGAU FOR 1900.

Once more enthusiastically interested spectators will be enabled to witness the remarkable representation which at intervals of ten years has been produced in the modest village of the Bavarian Tyrol, which on these occasions entertains many thousands of visitors. I have heard it asserted that the late unfortunate King of Bavaria had serious intentions of suppressing the Passion Play on the grounds that the enormous crowds of spectators from all corners of Europe and America tended to demoralise the simple peasants, and to awaken in them a greed for gold and notoriety, which in times past had no part in their childlike simplicity and unstudied devotion.

### The Origin of the Passion Play.

The Oberammergau dramatic representation is not a survival of a mediæval mystery, or miracle play, but took its rise from a vow made by the inhabitants in 1633 to represent our Lord's sufferings and death every ten years, with the hope of arresting the plague then raging fiercely in the valley. As a seal to this vow they erected a huge cross on an adjacent mountain peak, which retained its position till the winter of 1890, when, in a furious storm, it was blown down and shattered into fragments. A new one has been placed in nearly the same spot, but a little lower down the mountain. The original words were probably written by the monks of Ettal in their monastery close by, and doubtless they also superintended all arrangements. Clear information on these points is wanting, but in the beginning of the present century it is known certainly that all existing arrangements were remodelled by the parish priest when the Oberammergau Play obtained exemption from the general suppression of such exhibitions by the Government. The music was composed by Rodus Dedler, the village schoolmaster, in 1814.

### Present Arrangements.

I rejoice greatly in having seen the play before the covered theatre was built. In 1890 almost all the action took place in the open air, and resembled nothing so much as the Greek play at Bradfield College, which takes place in a grand natural amphitheatre. In '90 the Bavarians used a small stage, certainly, but the main part of the performance was outside in the proscenium. As something like 700 actors are needed, it will be easily understood that a very large space is needed. A large new theatre has been erected, and I cannot but fancy that these sumptuous accessories will spoil the effect somewhat; but what one loses in primitive picturesqueness, one doubtless gains in solid comfort. The performances occur every Sunday from May 20th to September 30th, with supplementary ones in the week if necessary. The villagers look upon the Passion Play as a solemn act of religion, and the acting is characterised by the greatest reverence. The most important parts are to a certain extent hereditary in certain families, and are distributed with a strict regard to moral character. It is considered almost a disgrace not to be allowed to take some part, however humble, in the play, and it is the dearest wish of every parent in Oberammergau that a son or daughter may in some happy year attain the supreme honour of representing the Christus or the Madonna. During the years between the performances the villagers are carefully drilled in dramatic performances by the parish priest, schoolmaster, &c., and no outside aid of any kind is sought.

### The Play Itself.

All the principal performers attend Mass early on the Sunday morning, and at half-past eight the play commence. There is an interval of an hour and a-half at mid-day, after which the drama continues till six p.m. Each scene in the history of our Lord is first typified by one from the Old Testament. The extremely exhausting effect produced upon the principal actor will prevent Josef Mayr from again taking the part, as he is now 52 years old. Moreover, he met with a serious accident some few years ago. It is not known who will be his successor, though rumour points to Johan Rundl, who represented St. John in 1890; the purity and calm of his expression makes him peculiarly suited to the honour, if his physical strength is equal to the strain. Rosa Lang will again take the part of the Madonna; in 1890 her father, the Burgomaster, was Caiaphas. Since then he is dead and his wife also, and Rosa is left alone.

### Accommodation.

This is so limited that it is necessary to make all arrangements far in advance. Messrs. Cook, whose information is reliable, puts the number able to find quarters in the village at 4,000. I should recommend all intending visitors to book early in the spring. I have no space to give information this week as to the various means of reaching Oberammergau, nor have I yet been able to obtain all information from agents, railways, &c. As soon as this is forthcoming I will say more on the subject. So far I see nothing so cheap and comfortable as Dr. Lunn's parties, which include a twenty days' tour for twenty-four guineas.

### Hints to Travellers.

I am going to give you a pattern for making a light and commodious knapsack for Continental use. Buy at any good furniture shop one yard of Chinese matting for floor covering; it is very fine, smooth, and close, and of an unobtrusive appearance. Cut a piece 30 in. deep by 12 wide. Cut two side pieces 11 in. deep by 3½ wide. Line all with pink jaconet, bought at the chemists; bind all pieces with coat binding. Sew four loops at convenient distances at the back of the knapsack to pass straps through, two loops to each strap. When you have fitted in the 11 in. side pieces, beginning at one end you will find you have a convenient flat at the top for covering well over and excluding dust. The whole to be fastened with cloak hooks and eyes, the hooks to be on the body of the knapsack and the eyes on two pieces of elastic on the flap. If the person is small who is about to construct this useful article I should cut the width only 10½ in. instead of 12, but the matter of size must always be settled by the individual. One of 12 by 11 will be found large enough to carry all that is necessary for a night's use, and is most convenient. When empty it only weighs a few ounces.

---

### TRAVEL NOTES AND QUERIES.

North Germany (Enquirer).—Hanover is considered an excellent place in which to study German. You say you know none. As you are not alone, I should recommend you to take up your abode in an entirely native hotel (I can tell you of some), by this means you will hear nothing of your own language, whereas if you go to an ordinary hotel frequented by different nationalities you will find the waiters all speak English more or less well. Fare to Hanover viâ Hook of Holland, first class, £2 14s. 8d.; second class, £1 17s. 6d.; viâ Harwich and Antwerp, first class, £3 17s. 9d.; second class, £2 13s. 6d.

Florence (Student).—There are several pensions, quiet and reasonable in terms, that would meet your views. Let me know if you really mean to go there. Very good accommodation may be had from 6 lire, which is 5s. per day. Your not knowing the language is a serious drawback, for the fashionable art teachers who can speak English charge accordingly, whereas could you speak Italian, however imperfectly, you could have cheap lessons from the humbler teachers, who are equally good in everything but fashion. You say you are not going till next autumn; in that case why not go in for six months' instruction in conversational Italian in the Gouin School, which undertakes to make you able to hold your own in Italy in six months?

Eaux Bonnes (Letty).—I should not go there until June, that is the ideal month, though May is very agreeable. Living dear. Journey, first class, viâ Dieppe, approximately, £6; and second, £4 5s.

